# Chemical Profiling of Sea Fennel (*Crithmum maritimum* L., Apiaceae) Essential Oils and Their Isolation Residual Waste-Waters

**DOI:** 10.3390/plants12010214

**Published:** 2023-01-03

**Authors:** Olivera Politeo, Marijana Popović, Maja Veršić Bratinčević, Kristina Kovačević, Branimir Urlić, Ivana Generalić Mekinić

**Affiliations:** 1 Department of Biochemistry, Faculty of Chemistry and Technology, University of Split, R. Boškovića 35, HR-21000 Split, Croatia; 2 Department of Applied Science, Institute for Adriatic Crops and Karst Reclamation, Put Duilova 11, HR-21000 Split, Croatia; 3 Department of Food Technology and Biotechnology, Faculty of Chemistry and Technology, University of Split, R. Boškovića 35, HR-21000 Split, Croatia

**Keywords:** sea fennel, essential oils, waste-water, phenolic compounds, GC-MS, HPLC

## Abstract

Sea fennel (*Crithmum maritimum* L.) is a wild-growing halophyte used in cuisine, traditional medicine or cosmetic products for its beneficial nutritive value and pleasant sensory characteristics. This study aimed to investigate sea fennel essential oils (EOs) from different parts of the plant (flowers, leaves and stems) and the corresponding hydrodistillation by-products (residual water) to validate their potential use and application in different industries. EOs were analyzed by gas chromatography coupled with mass spectrometry (GC-MS), while the phenolic profile of the residual water was analyzed by high-performance liquid chromatography (HPLC) and spectrophotometric methods. The EO analysis confirmed the presence of 14 compounds, dominated by sabinene (from 42.55 to 51.47%) and limonene (from 36.28 to 43.58%), while among the 12 detected phenolics, chlorogenic acid and its isomers (cryptochlorogenic and neochlorogenic acid) were found in the highest concentrations. Total phenolic, flavonoid and tannin contents were concentrated in the order flowers > leaves > stems. Although the sea fennel samples showed differences in chemical profiles, overall they were rich in bioactive compounds with relatively high amounts of key compounds with already proved good biological properties, especially in waste-water, indicating great potential for re-use in accordance with green processing technology trends.

## 1. Introduction

As more and more irrigated land has been affected by salinity in recent decades, special interest has been devoted to halophytic species. Halophytes are naturally salt-tolerant plants, and one of the most widespread and investigated edible halophytes is *Crithmum maritimum* L., commonly known as sea fennel or marine parsley. One of the interesting features of this plant is that it is the only species of the genus *Crithmum* within the family *Apiaceae*. Sea fennel can be found along the coasts of the Mediterranean and Black Seas and Atlantic Ocean. Despite its extremely valuable chemical composition, it is still highly underutilized and undervalued. This wild-growing aromatic fleshy herb is consumed in most Mediterranean countries due to its distinguishing sensory attributes in terms of taste, odor and color [1,2]. Numerous studies investigated sea fennel as a low-cost raw material with great nutritional value and functional properties to obtain natural, bioactive and health-promoting food ingredients [3,4]. Sea fennel is rich in various bioactive compounds with a recognized spectrum of different positive properties that have been investigated in previously published studies, such as antioxidant [3,4,5,6,7,8,9,10,11,12,13,14], antibacterial [6,7,10,13,15,16,17,18], antifungal [5,10,17,18], cytotoxic [4,18], anticancer [19], anti-inflammatory [4,5], antimutagenic [17], cholinesterase inhibitory [8,9], vasodilatory [9], and anti-parasitic [20] properties. In most of these studies, no matter if the authors were investigating sea fennel EO or non-volatile extracts, the dominant compounds in sea fennel isolates were the main contributors of the evaluated activity. Due to sea fennel’s valuable nutritional composition as well as these numerous reported positive biological properties, it is not surprising that this plant has been proposed as a new, valuable industrial cash crop halophytic species and a candidate for sustainable biosaline agriculture [21,22,23,24].

Herbs and aromatic plants, with their volatile metabolites, namely essential oils (EOs), are of great industrial interest due to their positive organoleptic and biological properties. EOs are mixtures of volatile components, mostly isolated from plant materials by distillation and are widely used in the perfume and cosmetics industry, but also in the food and pharmaceutical industries. In the case of hydrodistillation, the plant material is in direct contact with water, so after the EO isolation, high amounts of residual water with various non-volatile but usually extremely valuable and bioactive components remain. The conversion of EO isolation by-products into value-added formulations is a fundamental step for a green production technology, as they can be used in line with the principles of a sustainable circular economy. Given the current trends in the processing industry towards zero waste production and by-product recycling, it is, therefore, not surprising that various studies have addressed this issue [5,24,25,26,27]. 

Plants belonging to the botanical family Apiaceae are among the most investigated essential-oil bearing plants, and in recent decades, significant interest has been devoted to sea fennel EOs due to their high content and pleasant aroma characteristics. This plant is also characterized by its valuable nutritional content and high EO yield, which is why in the last few years the number of studies and publications about it has increased exponentially. However, only a limited number of studies (only one to the author`s knowledge) deal with the characterization of sea fennel EO isolation by-products [6]. In this regard, the aim of this work was to gain insight into the chemical profile of sea fennel EOs isolated from different plant parts (flower, stems, and leaves) and to analyze the phenolic potential of the corresponding residual water that is one of the main by-products of oil isolation. 

## 2. Results and Discussion

Sea fennel is a wild-growing plant with a salty taste and strong aromatic notes, which is why it is widely used in cuisine (as a spice or appetizer), in traditional medicine and/or in various cosmetic products [1,2]. Its pleasant sensory attributes, and especially its interesting flavor, which is usually described as a combination of common fennel, celery, and green citrus peel aroma, followed by a strong aftertaste, are the reasons why numerous studies have recently investigated sea fennel EO and its constituents [6,7,8,15,28,29,30,31,32].

The volatile aromatic substances of interest from various plant materials are usually isolated by solvent extraction, steam distillation or hydrodistillation. The first procedure is more time-saving and efficient, but has the disadvantage of producing large amounts of contaminating wastes (solvents) that must be properly disposed. Steam distillation and hydrodistillation, on the other hand, require greater energy inputs and are usually time-consuming, but no chemicals are needed, making them environmentally friendly. The final products obtained by these two methods are EOs. During steam distillation and hydrodistillation, volatile substances are isolated in the rising vapors of water and lipid droplets, and the final products after condensation are essential oils and aromatic water, usually called hydrolate. The second important liquid by-product of the EO extraction by hydrodistillation is non-distilled aqueous phase or waste-water (often also called decoction) [25,27]. The chemical composition and EO yield vary considerably depending on different biotic and abiotic factors, among which the plant part used as raw material, the plant’s developing stage, genetic and environmental factors, harvest season and location, and the isolation method used are among the most important [26]. In this study, the EOs obtained from different sea fennel plant parts, as well as residual waste-waters obtained during hydrodistillation, were chemically characterized.

### 2.1. Chemical Composition of Sea Fennel EOs

**Figure 1:**
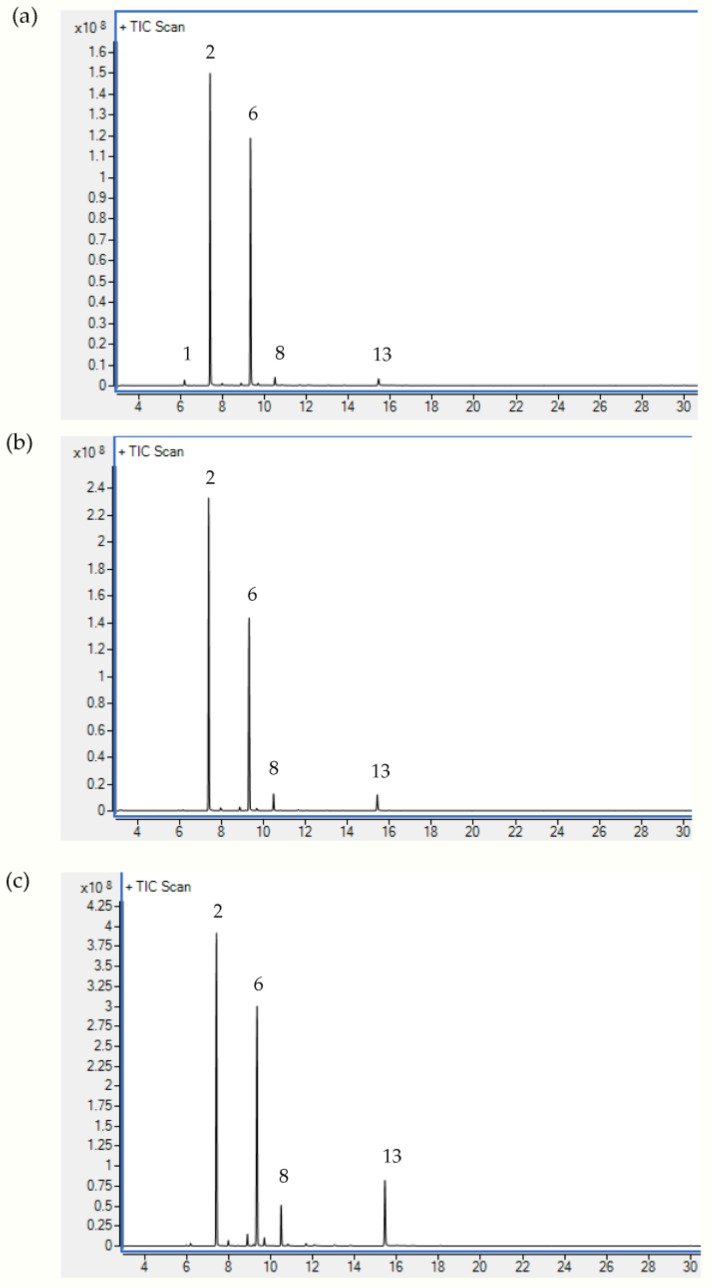
GC-MS total ion chromatogram of sea fennel essential oil obtained by hydrodistillation of (**a**) flower, (**b**) leaf, and (**c**) stem. Peak assignments are given in Table 1.

The components of sea fennel EOs isolated from different plant parts (flowers, leaves, stems) by conventional hydrodistillation in a Clevenger-type apparatus were characterized by gas chromatography coupled with mass spectrometry (GC-MS). As a result, 14 different compounds were detected, which differed in their presence and amount in the samples studied (Table 1, Figure 1). Differences were also obtained in the oil yield. The highest amount was obtained from the sea fennel flowers (1.35%), while more than 2-fold lower amounts were detected in the stems and leaves. The results of our previous study also showed the highest oil content in the flowers, followed by the leaves and significantly lower content in the stems [9], the same as in the study by Pateira et al. [28] on sea fennel from Portugal.

As shown in Table 1, the dominant components in all samples were non-oxygenated monoterpene compounds (ranging from 88.79% in EO from stem to 94.77% in flower EO samples). Among compounds from this chemical class, the highest amounts of sabinene (from 42.55 to 51.47%), limonene (from 36.28 to 43.58%) and *γ*-terpinene (from 2.79 to 5.28%) were detected. Among oxygenated monoterpenes, terpinen-4-ol was detected in all samples, with high amounts found in stems (10.35%). The presence of a high concentration of *α*-pinene was confirmed in flowers (1.76%) and of (*E*)-*β*-ocimene in flowers (0.77%) and stems (1.15%), while leaves (0.89%) and stems (0.80%) were rich in *β*-pinene. 

Differences between the obtained and previously published results on Croatian sea fennel could be observed. The samples from our previous study were rich in limonene (57–74%), *γ*-terpinene (5–14%) and sabinene (8–13%) [9], while limonene (58%), sabinene (26%), terpinene-4-ol (5%) and *γ*-terpinene (3%) were the dominant compounds in samples analyzed by Kulišić Bilušić et al. [33].

In addition, previous studies highlighted a high geographic variability in the major volatiles of sea fennel. According to Pateira et al. [28], two different chemotypes of sea fennel EO regarding the content of dillapiole can be distinguished: chemotype I with a content of 15–17% and chemotype II with 0–6%. Renna [21] reviewed the chemistry of sea fennel essential oils based on the results of different studies [6,7,8,15,28,29,30,31,32] and pointed out the intra-specific variability in sea fennel EO composition and yield, which can vary significantly depending on the plant geographic origin, life-cycle stage, collecting period, plant part, etc., so that different chemotypes can be distinguished: aromatic monoterpenes-type, monoterpene hydrocarbons-type, phenylpropanoids-type and their intermediate forms.

Since dillapiole was not detected in previous studies of Croatian sea fennel [9,34], as well as in the present study, we can conclude, in agreement with Pateira et al. [28], that Croatian sea fennel belongs to chemotype II, and according to the conclusions of Renna [21], it belongs to the monoterpene hydrocarbons essential oil chemotype. Sea fennel EOs from the studies of Baser et al. [29], Jallali et al. [6], Nabet et al. [10] and Alves-Silva et al. [5] were also mainly composed of monoterpene hydrocarbons and oxygenated monoterpenes with the reported dominance of limonene [15,16,21,28,31,33,34]. Baser et al. [29] reported that the main EO constituents of sea fennel from Turkey were sabinene (26.9%) and limonene (24.2%), followed by *γ*-terpinene and terpinen-4-ol, while limonene (22.3%), *γ*-terpinene (22.9%) and thymol methyl ether (25.5%) were the main EO constituents reported by Ruberto et al. [16]. In the study by Özcan et al. [31], monoterpenes were the dominant fraction (from 89.0 to 99.6%), and the main detected components were *γ*-terpinene (32–36%), *β*-phellandrene (21–22%), and sabinene (9–13%). In their later study, they found sabinene (22.3%), limonene (12.1%), *β*-phellandrene (10.3%), (*Z*)-*β*-ocimene (8.6%), *α*-pinene (7.1%), *γ*-terpinene (28.4%), and terpinene-4-ol (2.6%) in the samples studied [31]. 

### 2.2. Chemical Composition of Residual Waste-Water from Sea Fennel EO Isolation by Hydrodistillation

Hydrodistillation with a Clevenger-type apparatus is one of the most widely used extraction methods for EO isolation from plant material that is in direct contact with water (plant material soaking). This method is based on the application of high temperatures (boiling point of water), and the vapor mixture of water and essential oil is condensed by indirect cooling. However, the extraction parameters described, especially the extended isolation time and elevated temperatures, may lead to losses or chemical modifications of the components originally present in the plant material. It is well known that various phytochemicals, especially phenolics are highly susceptible to degradation and that different factors affect their stability, such as the presence of oxygen, high temperatures, the influence of the solvents, light, enzymes, proteins, metal ions, etc. [35]. Moreover, this method is time-consuming (the extraction time is around 3 h) and requires high energy consumption. Despite the very aggressive extraction parameters and due to the characteristics of the extraction process, different compounds are extracted in the water that remains in high quantities after the process. Although this residual waste-water is usually discarded, according to different studies, it is still a valuable source of a spectrum of non-volatile phytochemicals with beneficial biological properties [5]. 

Previous studies and revisions pointed out the richness of sea fennel in biologically active and nutritionally valuable compounds such as phenolic compounds [3,4,5,8,10,12,14,19], vitamin C [4,12], minerals (mainly calcium) [3,10,14,36,37,38], organic acids [36], carotenoids [10], carbohydrates [36,37], fatty acids, proteins, dietary fibers [36], etc. Among the constituents that sea fennel contains, phenolic compounds are the most widely investigated because they are secondary plant metabolites with a wide range of structures and positive biological functions. Numerous previous studies also confirmed the presence of valuable phytochemicals (from Table 2) in sea fennel samples as the main bioactive compounds [4,5,7,8,9,11,12,22,36].

**Figure 2:**
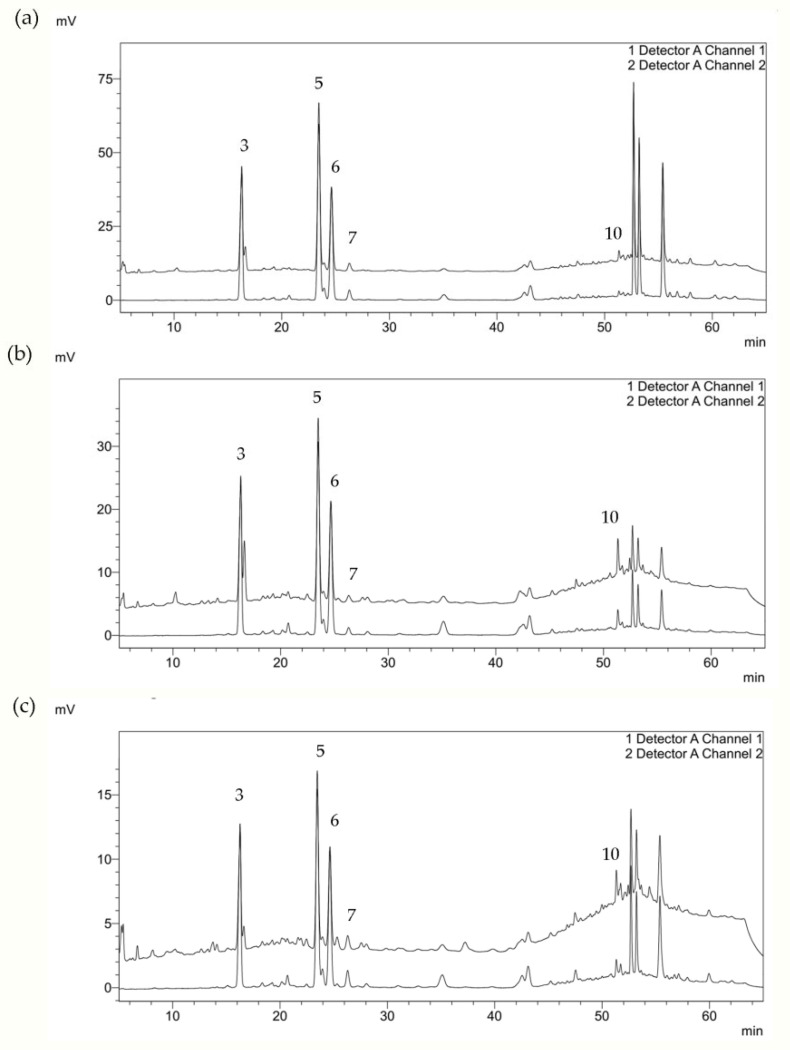
HPLC chromatogram of residual water after sea fennel EO hydrodistillation of (**a**) flower, (**b**) leaf, and (**c**) stem at 220 nm (upper) and 320 nm (lower) line. Peak assignments of identified and quantified phenolic compound are given in Table 2.

Phenolic compounds detected in residual water that remains after EO extraction from different sea fennel plant parts are presented in Table 2, and corresponding chromatograms are shown in Figure 2.

According to the presented results, chlorogenic acid was the dominant compound in all samples (from 4.48 to 17.69 mg/g), with the highest amount detected in the flower extract. Meot Duros and Magné [37] reported that sea fennel is one of the species with the highest chlorogenic acid content within the Apiaceae family. The presence of this compound in the highest concentration in sea fennel was also reported in our previous studies [8,9] and by other authors [3,4,5,10,11,12]. Among other compounds, two isomers of chlorogenic acid were found in high concentrations: cryptochlorogenic acid (4-*O*-caffeoylquinic acid) and neochlorogenic acid (5-*O*-caffeoylquinic acid). Again, the highest amounts were found in the flower samples, and the lowest in the stems. This is in agreement with the results of Pereira et al. [3] and Souid et al. [17], who also found the highest concentrations of chlorogenic acid, cryptochlorogenic acid and neochlorogenic acid in leaf samples of sea fennel.

Sánchez-Faure et al. [36] reported that the dominant phenolics in sea fennel are hydroxycinnamic acids derived from the esterification of cinnamic acids such as caffeic, ferulic, and *p*-coumaric acids with quinic acid, while Siracusa et al. [11] concluded that chlorogenic acid and its isomers are almost the sole class of phenolics in sea fennel infusions. In their study on waste-water that remains after isolation of sea fennel EOs, Alves-Silva et al. [5] reported that the major phenolic acids were chlorogenic acid, cryptochlorogenic acid and neochlorogenic acid, but that *p*-coumaric acid, 3,5-dicaffeoylquinic acid isomer, 3,4-dicaffeoylquinic acid and 4,5-dicaffeoylquinic acid were also found.

Vanillic acid, caffeic acid and cinnamic acid were detected in the ethanolic extracts of sea fennel from our previous study [8]. Pereira et al. [3] detected only the presence of *p*-hydroxybenzoic acid from the group of hydroxybenzoic acids. This compound was also found in our study (from 0.11 mg/g in flowers to 0.39 mg/g in leaves), as well as small amounts of gallic acid. Özcan et al. [31] reported the presence of gallic acid, protocatechuic acid and *p*-coumaric acid in high concentrations in their study. Souid et al. [17] detected *trans*-ferulic acid, and Kadoglidou et al. [12] confirmed the presence of protocatechuic acid, caffeic acid, and *p*-coumaric acid (although in negligible amounts compared to the dominant chlorogenic acid and its isomers). This is in agreement with our results, where higher concentrations of caffeic acid (0.52 mg/g) and ferulic acid (0.30 mg/g) were found in flowers, and *p*-hydroxybenzoic acid (0.39 mg/g) in leaves.

Among flavonoids, quercetin, rutin and myricetin were found (Table 2). High concentrations of quercetin were detected in leaves (2.01 mg/g), while concentrations in flowers and stems were lower. Rutin and myricetin were found in the highest amounts in the flower sample, of 0.53 and 0.44 mg/g, respectively. Piatti et al. [22] also confirmed the presence of rutin in ethanolic extracts of sea fennel, as did Aleman et al. [4] and Kadoglidou et al. [12], who reported that the major flavonoids in sea fennel were generally quercetin derivatives.

**Figure 3:**
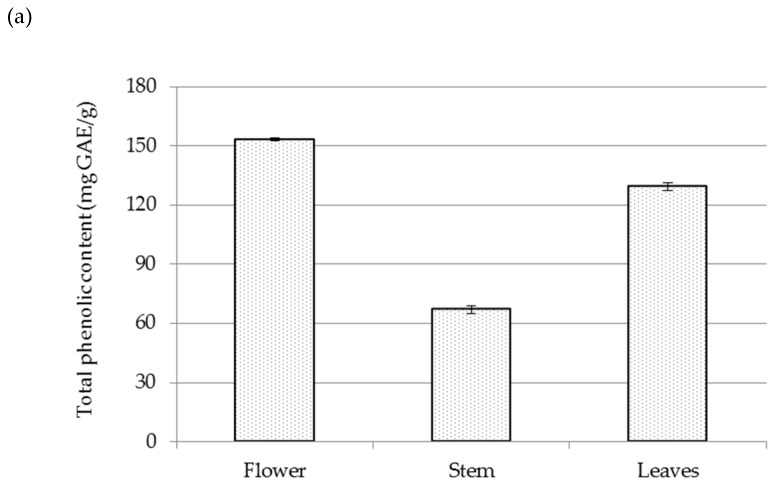

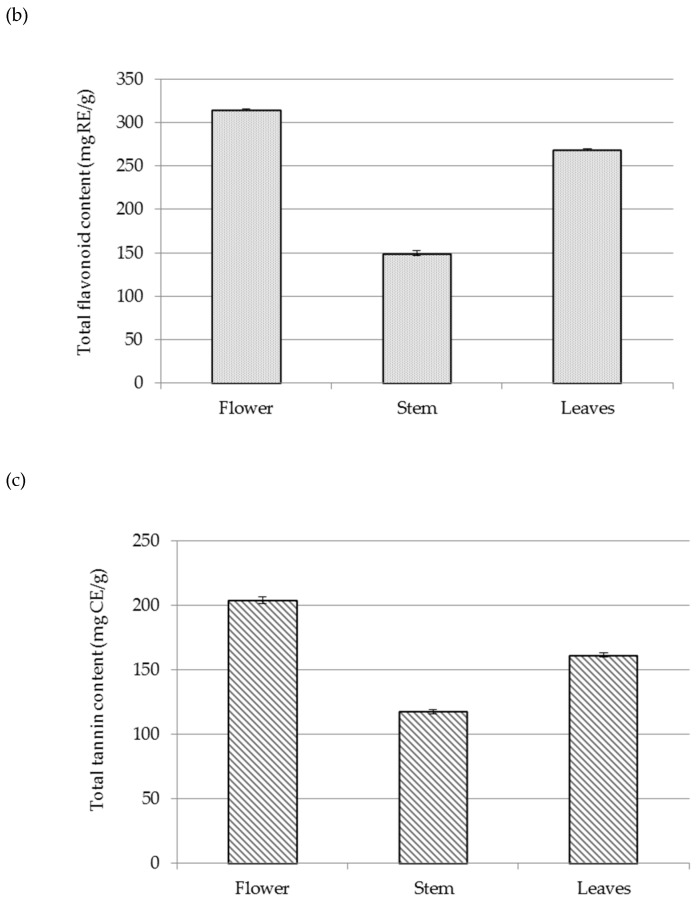
Total phenolic content (**a**) in mg gallic acid equivalents (GAE)/g, total flavonoid content (**b**) in mg rutin equivalents (RE)/g, and total tannin content (**c**) in mg catechin acid equivalents (CE)/g in sea fennel samples.

Sea fennel residual-water extracts were also screened for total phenolic content (TPC), total flavonoid content (TFC) and total tannin content (TTC) by spectrophotometric methods (Figure 3), and the results are expressed per g of dry extract. From the presented results, it can be seen that all detected compounds were present in samples in the following order: flowers > leaves > stems. The TPC ranged from 61.7 to 153.3 mg GAE/g, TFC ranged from 149.8 to 314.8 RE/g, and TTC ranged from 117.5 to 204.0 CE/g.

Comparison of the obtained results with previously published ones is extremely difficult due to differences in the origin of the plants and other abiotic factors affecting them, as well as in the applied extraction parameters such as type of solvent, ratio of solid-to-solvent, extraction temperature and time, application of new techniques, etc. Maleš et al. [34] studied Croatian sea fennel from different geographical locations and at different growth stages and the results obtained showed variations depending on of the growing site and growth stage. The highest TPCs, TFCs and TTCs were found in the samples collected before flowering. Houta et al. [7] also investigated phenolics from different sea fennel plant parts and reported higher content of all mentioned phenolic sub-groups in methanolic extracts of the stems compared to the flowers and leaves, which is in contrast to our results. However, the authors pointed out the importance and influence of the extraction parameters such as the extraction solvent used, the temperature applied, extraction duration, extraction mode, etc. Pereira et al. [3] reported the phenolic content in sea fennel infusions and decoctions from different plant parts. In their study, the highest values for TPC, TFC and condensed tannins were found in leaves, followed by flowers and stems [9]. 

Usually, for the extraction of the phenolics from plant material, alcoholic aqueous mixtures are used, and the temperature is usually low in order to prevent degradation of thermolabile compounds. Additionally, the extraction is often assisted by some other technique (e.g., ultrasonication, microwaves) to increase the efficiency. In our study, water was used as an extraction solvent, and the applied extraction parameters were extremely aggressive (temperature above 100 °C, extraction time 2.5 h), which significantly influenced the obtained results. However, in spite of this, the residual waste-water that remains after the EO isolation is still a valuable source of phenolic compounds, especially that from flowers and leaves.

## 3. Materials and Methods

### 3.1. Plant Material and Reagents

The plant material, the flowering aerial parts of wild-grown sea fennel (*Crithmum maritimum* L.), was collected in Central Dalmatia (43°39′44″ N 15°56′40″ E, Croatia) in September 2022 at the plant full-flowering stage (approx. 1 kg from more than 10 increments from the same location, of around 10 m^2^). Prior to the isolation of essential oils, the material was air-dried (15 days at room temperature), and after drying, was divided into plant parts, namely, flowers, leaves and stems. 

Voucher specimens of the plant material were deposited in the herbarium of the Department of Biochemistry, Faculty of Chemistry and Technology, University of Split. All chemicals, reagents and solvents used were of adequate analytical grade and were purchased from Kemika (Zagreb, Croatia) and Sigma-Aldrich (St. Louis, MO, USA).

### 3.2. Isolation of Essential Oils and Preparation of Residual-Water Extracts

Sea fennel EOs from the different plant sections (flowers, stem, and leaves) were isolated from the dry plant material by hydrodistillation in a Clevenger-type apparatus for 3 h according to the procedure described by Kulišić-Bilušić et al. [33]. The isolated EOs were dried over anhydrous sodium sulfate, and the oil yield was calculated based on the dry plant material. 

The waste-water that remained after oil extraction was cooled, filtered and freeze-dried, and the crude extracts obtained were dissolved in water (at a concentration of 10 mg/mL) and used for further analysis. 

### 3.3. Gas Chromatography-Mass Spectrometry (GC-MS)

The gas chromatography system used for analysis of the sea fennel EOs consisted of a gas chromatograph (model 8890 GC) equipped with an automatic liquid injector (model 7693A) and a tandem mass spectrometer (MS/MS) (model 7000D GC/TQ), all from Agilent Inc. (Santa Clara, CA, USA). Separation was performed on the HP-5MS UI (30 m × 0.25 mm i.d., stationary phase layer thickness 0.25 μm) (Agilent Inc.) column. The column temperature program was set at 60 °C for the first 3 min, then heated to 246 °C at 3 °C/min and maintained for 25 min isothermally. Helium was the carrier gas, the flow rate was 1 mL/min, and inlet temperature was 250 °C, while the sample injection volume was 1 µL. MS conditions were: ionization energy 70 eV; ion source temperature 200 °C; the temperature of the quadrupoles 150 °C. Individual peaks were identified by comparing their retention indices (relative to C_8_-C_20_ *n*-alkanes for HP-5MS UI) to those of authentic samples and the literature, as well as by comparing their mass spectra with the Wiley 7 MS library (Wiley, NY, USA) and the NIST02 (Gaithersburg, MD, USA) mass spectral database. The percent compositions of the samples in Table 1 were calculated from the peak areas using the normalization method (without correction factors) as averages of three injections.

### 3.4. High Performance Liquid Chromatography (HPLC)

Phenolic compounds were separated, identified and quantified using a Shimadzu Nexera LC-40 (Shimadzu, Kyoto, Japan), equipped with a UV-VIS detector, using Phenomenex C18 reverse-phase column (250 mm × 4.6 mm, 5 μm; Torrance, CA, USA). The flow rate was 1 mL/min, and the temperature was 35 °C. The mobile phase A was ultra-pure water/85% *o*-phosphoric acid (99.8/0.2, *v/v*), whereas the mobile phase B was methanol/acetonitrile (1/1, *v/v*). The total run time was 65 min using a concentration gradient as follows: initially 4% B; 16 min 15% B; 50 min 35% B; 62 min 4% B and held until 65 min. The detected compounds were identified by comparing their retention times and absorption maximums at 220 and 320 nm with those acquired for corresponding standards and by sample spiking, while they were quantified using external standard calibration curves. The concentrations of the compounds are expressed as mg of compound per gram of dry extract (mg/g).

### 3.5. Spectrophotometric Measurements of Main Phenolic Groups

The obtained residual-water extracts were analyzed spectrophotometrically for total phenolics (TPC), flavonoids (TFC) and tannins (TTC) using a SPECORD 200 Plus, Edition 2010 (Analytik Jena AG, Jena, Germany). 

Total phenolics were determined by the Folin–Ciocalteu method [39]. Results are expressed as mg gallic acid equivalents per gram of extract (mg GAE/g).

Total flavonoids were determined according to the method proposed by Yang et al. [40]. The results are expressed in mg of rutin equivalents per gram of extract (mg RE/g). 

Tannins were determined according to the procedure reported by Julkunen-Titto [41], and the results are expressed in mg catechin equivalents per gram of extract (mg CE/g).

### 3.6. Statistical Analysis

The results are expressed as mean (±standard deviation) of a set of three independent measurements. Statistical analysis was performed using GraphPad Instat3 (GraphPad Software, San Diego, CA, USA). 

## 4. Conclusions

This study aimed to chemically characterize the sea fennel EOs and their isolation by-products to gain insight into the potential significance and value of sea fennel (flowers and leaves) and the plant parts that are commonly discarded and/or treated as waste after the crop production (stems) and/or oil extraction (waste-water). The presented results proved once again that Croatian sea fennel belongs to the sea fennel chemotype II due to the absence of dillapiol and to the chemotype of monoterpene hydrocarbon essential oil due to the dominance of this chemical subclass in the EOs. It is well known that the chemical composition of EO varies considerably depending on different biotic and abiotic factors, but this study confirmed the variability among the plant parts used. Moreover, despite the very aggressive EO isolation parameters (high temperatures, long extraction time), the residual waste-water that remained in large quantities after the EO isolation still contained high concentrations of biologically important compounds, especially chlorogenic acid and its isomers. Therefore, sea fennel by-products and waste materials from the plant’s cropping and processing should promote additional industrial interest, as they can be used as suitable raw materials for the development of food, pharmacological or cosmetic ingredients and products, and future studies should take in consideration these findings and focus on their potential application in different products. In addition, their reuse will reduce the environmental impact of by-product disposal, which is currently one of the most important challenges facing the global economy. 

## Figures and Tables

**Table 1:** GC-MS chemical composition of sea fennel essential oils from different plant parts.

No.	Component	RI	Flower	Leaf	Stem	Mode of Identification
	*Monoterpene Hydrocarbons*					
1.	*α*-Pinene	938	1.76	0.08	0.35	RI, MS
2.	Sabinene	977	44.94	51.47	42.55	RI, MS
3.	*β*-Pinene	993	0.07	0.89	0.80	RI, MS
4.	α-Terpinene	1019	0.86	0.98	1.47	RI, MS
5.	*p*-Cymene	1028	n.d.	0.09	0.27	RI, MS
6.	Limonene	1032	43.58	36.28	36.45	RI, MS
7.	(*E*)-*β*-Ocimene	1042	0.77	0.07	1.15	RI, MS
8.	γ-Terpinene	1062	2.79	3.49	5.28	RI, MS
10.	Terpinolene	1090	n.d.	0.37	0.47	RI, MS
	*Oxygenated Monoterpenes*					
9.	*cis*-Sabinene hydrate	1070	0.11	0.10	0.38	RI, MS
11.	*trans*-Sabinene hydrate	1098	0.08	n.d.	0.15	RI, MS
12.	*trans*-*p*-Menth-2-en-1-ol	1122	n.d.	0.10	0.13	RI, MS
13.	Terpinen-4-ol	1179	3.53	5.35	10.35	RI, MS
14.	*α*-Terpineol	1190	n.d.	n.d.	0.07	RI, MS
	Total identified (%)		98.49	99.27	99.87	
	Oil yield (%, *w/w*)		1.35	0.63	0.62	

n.d.—not detected; RI = retention indices on HP-5MS UI column; MS = mass spectra.

**Table 2:** HPLC phenolic composition (mg/g) of residual water extracts after essential oil extraction from different sea fennel plant parts.

No.	Component	Flower	Leaf	Stem
	*Phenolic acids*			
1.	Gallic acid	0.05 ± 0.02	0.04 ± 0.01	0.03 ± 0.03
2.	Protocatechuic acid	0.06 ± 0.00	0.08 ± 0.00	0.06 ± 0.00
3	Neochlorogenic acid (5-*O*-caffeoylquinic acid)	11.47 ± 0.09	6.56 ± 0.05	3.27 ± 0.02
4.	*p*-Hydroxybenzoic acid	0.11 ± 0.01	0.39 ± 0.16	0.23 ± 0.02
5.	Chlorogenic acid (3-*O*-caffeoylquinic acid)	17.69 ± 0.00	9.17 ± 0.05	4.48 ± 0.02
6.	Cryptochlorogenic acid (4-*O*-caffeoylquinic acid)	11.04 ± 0.01	6.16 ± 0.03	3.10 ± 0.01
7.	Caffeic acid	0.52 ± 0.00	0.18 ± 0.00	0.20 ± 0.00
8.	Ferulic acid	0.30 ± 0.01	0.13 ± 0.01	0.11 ± 0.00
9.	Sinapic acid	0.08 ± 0.01	tr	0.02 ± 0.00
	*Flavonoids*			
10.	Quercetin	1.55 ± 0.06	2.01 ± 0.03	0.97 ± 0.14
11.	Rutin	0.53 ± 0.01	0.47 ± 0.01	0.37 ± 0.02
12.	Myricetin	0.44 ± 0.02	0.09 ± 0.02	0.14 ± 0.08

tr—traces. The results are expressed as mean ± SD.

## Data Availability

Not applicable.
